# Peroxiredoxin 2: a potential biomarker for early diagnosis of Hepatitis B Virus related liver fibrosis identified by proteomic analysis of the plasma

**DOI:** 10.1186/1471-230X-10-115

**Published:** 2010-10-13

**Authors:** Ye Lu, Jie Liu, Chengzhao Lin, Haijian Wang, Ying Jiang, Jiyao Wang, Pengyuan Yang, Fuchu He

**Affiliations:** 1 Department of Chemistry, Fudan University, Shanghai, China; 2 Laboratory of Systems Biology, Institutes of Biomedical Sciences, Fudan University, Shanghai, China; 3 Department of Molecular Biology for Public Health, Shanghai Municipal Centers for Disease Control and Prevention, Shanghai, China; 4 Department of Gastroenterology, Zhongshan Hospital, Fudan University, Shanghai, China; 5 State Key Laboratory of Proteomics, Beijing Proteome Research Center, Beijing Institute of Radiation Medicine, Beijing, China

## Abstract

**Background:**

Liver fibrosis is a middle stage in the course of chronic Hepatitis B virus (HBV) infection, which will develop into cirrhosis and eventually hepatocellular carcinoma (HCC) if not treated at the early stage. Considering the limitations and patients' reluctance to undergo liver biopsy, a reliable, noninvasive diagnostic system to predict and assess treatment and prognosis of liver fibrosis is needed. The aim of this study was to identify biomarkers for early diagnosis of HBV related liver fibrosis.

**Method:**

Plasma samples from 7 healthy volunteers and 27 HBV infected patients with different stages of fibrosis were selected for 2-DIGE proteomic screening. One-way ANOVA analysis was used to assess differences in protein expression among all groups. The alteration was further confirmed by western blotting. Plasma levels of 25 serological variables in 42 healthy volunteers and 68 patients were measured to establish a decision tree for the detection of various stages fibrosis.

**Result:**

The up-regulated proteins along with fibrosis progress included fibrinogen, collagen, macroglobulin, hemopexin, antitrypsin, prealbumin and thioredoxin peroxidase. The down-regulated proteins included haptoglobin, serotransferrin, CD5 antigen like protein, clusterin, apolipoprotein and leucine-rich alpha-2-glycoprotein. For the discrimination of milder stage fibrosis, the area under curve for Prx II was the highest. Four variables (PT, Pre, HA and Prx II) were selected from the 25 variables to construct the decision tree. In a training group, the correct prediction percentage for normal control, milder fibrosis, significant fibrosis and early cirrhosis was 100%, 88.9%, 95.2% and 100%, respectively, with an overall correct percent of 95.9%.

**Conclusion:**

This study showed that 2-D DIGE-based proteomic analysis of the plasma was helpful in screening for new plasma biomarkers for liver disease. The significant up-expression of Prx II could be used in the early diagnosis of HBV related liver fibrosis.

## Background

Liver fibrosis is a middle stage in the course of chronic HBV infection, which will develop into cirrhosis and eventually hepatocellular carcinoma (HCC) if not treated at the early stage. The risk of developing cirrhosis depends on the degree of fibrosis (stage) and the degree of inflammation and necrosis (grade) in liver [1,2]. Although liver biopsy is currently recommended as the gold standard method of staging fibrosis in patients with chronic HBV, it has several disadvantages such as poor patient compliance, sampling error, limited usefulness for dynamic surveillance and follow-up. Considering these limitations and patients' reluctance to undergo a liver biopsy, there is a need for the development of novel noninvasive techniques to detect early liver damage. Several clinical studies have attempted to identify serological markers that rely on the measurement of substances participating in the generation of the liver extra cellular matrix. The current applications include hyaluronic acid (HA) [3,4], type IV collagen (CIV) [4], N-terminal propeptide of type III procollagen (PIIIP) [3,5], metalloproteinases [6], inhibitors of metalloproteinases [6], and transforming growth factor beta [7]. Although some of these markers have shown promise for the detection of advanced fibrosis, their sensitivities for detecting milder fibrosis are generally poor. Therefore, a reliable, noninvasive diagnostic system to predict and assess treatment and prognosis of liver fibrosis is needed.

The biomarkers mentioned above have been identified through a candidate approach (i.e. derived from knowledge of basic biology and pathophysiology insights). With recent advances in genomics and proteomics, biomarkers can now be identified by discovery (or hypothesis generation) strategies that are not limited by our existing biological knowledge. By comprehensively examining different protein expression profiles between normal and pathological or drug treated samples, proteomics may provide information on new biomarkers, disease associated targets and the process of pathogenesis. This technique has been extensively employed to investigate cancers and other diseases [8-17].

2-DE is a powerful technique capable of resolving several thousand proteins based on their isoelectric points in the first dimension and their sizes in the second dimension [18]. A fundamental improvement was the development of 2-D fluorescent difference in-gel electrophoresis (DIGE) [19], which has the ability to analyze multiple protein samples within one gel. This is achieved through covalent modification of each protein with structurally similar but spectrally distinct fluorphores (CyDye2, CyDye3, and CyDye5). On each gel, two samples and an internal standard comprising an equal amount of each sample within the study can be examined. This process reduces the gel-to-gel variation and allows more accurate and sensitive proteomic quantization [20,21].

In the present study, we employed the DIGE technology to identify plasma profiles of liver fibrosis-related proteins, and the further confirmation with western blotting and ELISA showed that Prx II was better than the current available markers in detecting milder fibrosis. The present findings demonstrate that proteomics is a powerful approach for the molecular characterization of liver fibrosis progression and the identification of novel markers for the diagnosis of liver fibrosis.

## Methods

### Human Samples

Plasma samples of 49 healthy volunteers and 95 patients with chronic hepatitis B virus infection were collected between 2004 and 2005 from Zhongshan Hospital, Shanghai and State Key Laboratory of Proteomics, Beijing Proteome Research Center, Beijing, with the approval of the Ethical Committee at Fudan University. In screening study, subjects were restricted to patients having the same G3 grade inflammation (moderate piecemeal necrosis in portal with severe focal cell damage in lobule) to minimize unrelated variables and six groups (healthy, S1-S4 fibrosis and cirrhosis) were enrolled (Table 1). In validation study, four groups (healthy, milder fibrosis (S1), significant fibrosis (S2 and S3), early cirrhosis (S4)) were enrolled to facilitate data analysis (Table 2).

**Table 1:** Clinical features of all the subjects for screening study

	Healthy	G3S1	G3S2	G3S3	G3S4	Cirrhosis
Number	7	5	5	5	7	5
Age (Mean ± SD)	33.3 ± 9.1	35.8 ± 12.0	34.4 ± 14.3	34 ± 10.4	38.5 ± 15.1	43.3 ± 10.1
Gender (Male/Female)	7/0	4/1	4/1	4/1	6/1	4/1

**Table 2:** Clinical features of all the subjects for validation study

	Healthy	Milder fibrosis	Significant fibrosis	Early Cirrhosis
Number	42	24	32	12
Age (Mean ± SD)	32.9 ± 9.2	37.4 ± 12.4	34.4 ± 11.7	38.5 ± 14.4
Gender (Male/Female)	42/0	22/2	28/4	10/2

All patients were measured for HBsAg, HBeAg and HBcAg for sure of anti-HBV positive. All patients were HBV-DNA positive (bDNA Assay 3.0, Bayer, Leverkusen). Patients with other hepatitis virus (HAV, HCV, HEV) infection, other liver diseases, HIV co-infections, and other malignomas or antiviral treatment were excluded. Liver biopsies were obtained from all patients. All samples ranged from 1.5 to 2.0 cm long, more than 1.0 mm thick and included at least 10 portal tracts. Classification of the fibrosis stages and inflammation grades was done according to Scheuer *et al. *[22]. The results of biopsy were interpreted by two pathologists independently.

### Preparation of plasma samples

Blood samples were collected in sodium heparin coated plastic tubes for the preparation of plasma. After centrifuged at 4,000 g for 10 min, the supernatant plasma were divided and stored in aliquots at -80°C until analysis. Samples were thawed only once for the study. Before 2-DE, plasma was pretreated to deplete albumin and IgG with the ProteoExtract Albumin/IgG Removal Kit (Calbiochem, Darmstadt, Germany) and desalted with the ProteoExtract Protein Precipitation Kit (Calbiochem, Darmstadt, Germany). All experiments were done according to the manufacture's instruction. Samples were dissolved in 100 μL of the DIGE lysis buffer (8 M Urea, 4% w/v CHAPS and 30 mM Tris) and adjusted to pH 8.5. Protein content was determined using the modified method of Bradford.

### DIGE Electrophoresis

For screening study, samples in the same group were pooled to decrease the individual differences. The same group of sample was labeled with either CyDye3 or CyDye5 and ran in two different gels to eliminate the effect of dyes. 50 μg protein of each group was labeled with 0.8 μL of CyDye3 or CyDye5 DIGE fluors minimal dyes (400 μM), respectively. After 30 min, the incubation was stopped by adding 1 μL of 10 mM lysine. The labeled samples were further diluted with an equal volume of the 2× sample buffer containing 8 M urea, 4% w/v CHAPS, 2% DTT, and 2% Pharmalyte pH3-10. The internal standard included 8.33 μg of each group (6 groups in total) labeled with CyDye2. Two different groups (CyDye3 and CyDye5) and the internal standard (CyDye2) were run per gel. The three labeled samples were mixed and the volume was adjusted to 350 μL with rehydratation buffer containing 8 M urea, 4% w/v CHAPS, 13 mM DTT, and 1% (v/v) IPG ampholytes pH 3-10. All gels, six in total, were processed and analyzed simultaneously. The first dimension was carried out on an IPGphor system (Amersham Biosciences) using pH3-10 IPG gel strips of 24 cm. The IEF was performed at 20°C under the following conditions: 12 h at 50 V; 30 min at 250 V; 30 min at 500 V; 1 h at 1000 V; 1 h at 2000 V; 1 h at 4000 V; 2 h at 8000 V and held at 8000 V until the total Vhr reached 80000 Vhr. After isoelectric focusing, the IPG strips were equilibrated for 10 min in a reduction buffer (6 M urea, 30% (v/v) glycerol, 0.5% w/v DTT, and 2% (m/v) SDS in 0.05 M Tris-HCl buffer, pH8.8) and subsequently alkylated for 10 min in an alkylation buffer containing 6 M urea, 30% (v/v) glycerol, 4.5% (w/v) iodoacetamide, and 2% (w/v) SDS in 0.05 M Tris-HCl buffer, pH8.8. The second dimensional separation was carried out on the custom-made 12% SDS-polyacrylamide gels and a Hoefer DALT electrophoresis system (Amersham Biosciences).

### Gel Image and Data Analysis

The gels were scanned using the Typhoon 9410 laser scanner (Amersham Biosciences) at three different settings (CyDye2, blue laser 488 nm and 520 bp 40 filter; CyDye3, green laser 532 nm and 580 bp 30 filter; CyDye5, red laser 633 nm and 670 bp 30 filter). Three images per gel were obtained (18 in total). The scanned images were analyzed using DeCyder 6.5 (Amersham Biosciences). Spots were automatically detected and visually checked for undetected or incorrectly detected spots. The protein spots detected in each image were automatically linked among the three images per gel. All gels were matched to a digitized reference gel, containing all the protein spots present in all six internal standard images. The intensity levels per image were normalized by dividing the spot volume through the total intensity of all the spots in the image and multiplying it by the average of the total spot intensity of all 18-gel images. Subsequently, the CyDye3 and CyDye5 labeled spot volumes were divided by the spot volume of the corresponding protein spot in the internal standard (CyDye2) image. The differences in spot ratios were analyzed by one-way ANOVA analysis and the Student's *t *test (assuming normal distributions and equal variance). One-way ANOVA was performed for the parameter of ''liver fibrosis''. The *P *value cut-off for ANOVA was 0.05. The proteins, found to be significant in the first step, were further analyzed with the *t *test between each paired groups. The *P *value cut-off for the *t *test was 0.05, and the fold change was 1.5.

### In-gel Digestion and MALDI-TOF MS

After scanning, the gel was stained by the MS accommodated silver staining method. For each gel spot, a biopsy punch was speared (Amersham Biosciences) and transferred to a 1.5 mL siliconikzed Eppendorf tube. Subsequently, the transferred gel spots were destained in a destaining solution (100 mM Na_2_S_2_O_3 _and 30 mM K_3_Fe (CN)_6_, V/V, 1:1). The destained gel slices underwent pre-reduction using 100% acetonitrile (HPLC grade), and gel slices were dried in a Speed-Vac. After dried, gel slices were incubated at 37°C for 12-16 h in an ABC buffer (50 mM ammonium bicarbonate, pH8.0) containing 0.1 mg/mL sequencing grade modified trypsin (Promega Biosciences, San Luis Obispo, CA). Peptide samples were mixed at a ratio of 0.5 μL matrix (R-cyano-4-hydroxytranscinnamic acid) and 0.5 μL sample, loaded onto a 96×2 samples plate (Corning, P/N V700813), and crystallized. The crystallized samples were analyzed using an Applied Biosystems 4700 Proteomics Analyzer. In addition, trypsin-digested myglobin was used as an external standard for the mass calibration. PMF and sequence data were matched by searching the Swiss-Prot database using the MASCOT engine (Matrix Science).

### Western blot analysis

For Western blot analysis, undepleted plasma proteins (20 μg) were loaded onto each lane, size fractionated by SDS-PAGE, transferred to PVDF membrane (Amersham Pharmacia Biotech), and blocked with PBS/5% skim milk/0.01% Tween 20 for 30 min at room temperature. Primary polyclonal antibodies (Abcam) diluted according to the manufacture's instructions in a blocking buffer were added, with subsequent incubation for 1 h with horseradish peroxidase-conjugated secondary antibodies (Abcam). Samples were washed and developed with ECL-Plus (Amersham Pharmacia Biotech).

### Serological analysis

The plasma levels of Prx II, CLU, HP, Apo AI, LN, CIV and PIIIP were detected by the double antibody sandwich ELISA assay according to the published method [23]. All antibodies used are from Abcom, Santa Cruz or Abnova. ELISA absorbance at 450/570 nm was measured to analyze the plasma protein levels semi-quantitatively. The plasma levels of HA were detected by RIA with the HA test kit, following the manufacture's instructions. Other clinical biochemical tests were done as a routine work in our laboratory on Hitachi 7600 Biochemistry Auto analyzer (Hitachi, Japan). Two independent researchers from our group performed all analyses blinded and in duplicate.

### Statistical Analysis

The SPSS 13.0 (SPSS Inc., Chicago, IL) was used to perform all statistical comparisons. All comparisons were two-tailed, and a *P *value < 0.05 was considered significant. Independent sample *t*-tests were used to analyze protein plasma differences among the various groups. ROC curves and AUCs were calculated, with 95% CIs. Classification tree was developed and the growing method is CHAID with a significant level of 0.05 for both splitting and merging.

## Results

### Quantitative comparison and identification of protein spots on DIGE gels

**Figure 1:**
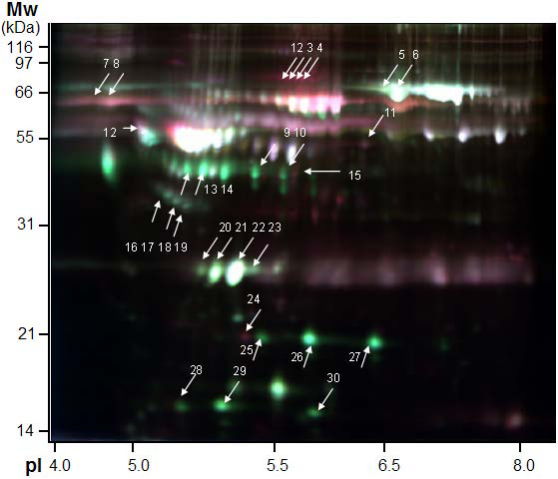
**False-colored DIGE gel image of plasma proteins from normal and patient groups**. Cy2 (blue) image of proteins from internal standard plasma; Cy3 (green) and Cy5 (red) image of proteins from plasma of different groups. The overlay images showed white spots containing proteins that have equal expression levels in each two group samples, red spots containing proteins with a higher expression and green spots containing proteins with a lower expression in the progressive phase of fibrosis. Spots for which the volume |ratio| ≥ 1.5 (*t *test) and *P *≤ 0.05 (one-way ANOVA) based on DeCyder software analysis were identified by MS. In some cases, different spots were identified as the same protein.

**Figure 2:**
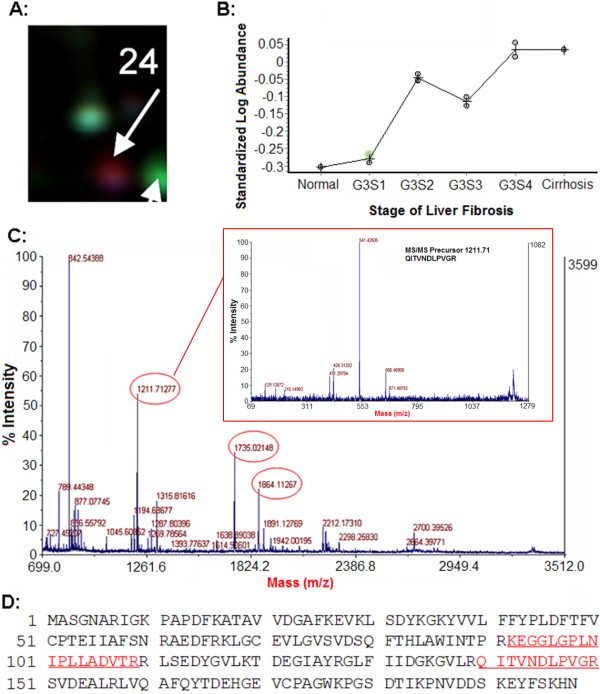
**Plasma Prx II levels were up-regulated with fibrosis progress**. A: Magnified region of DIGE gel image of Prx II. B: The patterns of the relative abundance alterations of Prx II in different groups. C: The MALDI-TOF MS map of Prx II, in which peptide peaks for further MS/MS identification are labeled out with mass value and the MS/MS map of peptide 1121.71 was shown. D: The amino acid sequences of Prx II, in which MS/MS matched peptide sequences, are underlined.

**Figure 3:**
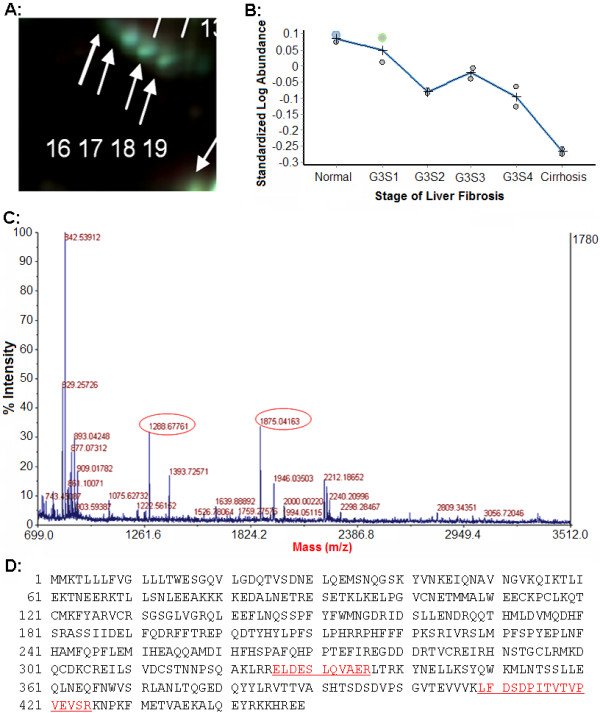
**Plasma CLU levels were down regulated with fibrosis progress**. A: Magnified region of DIGE gel image of CLU. B: The patterns of the relative abundance alterations of CLU in different groups. C: The MALDI-TOF MS map of CLU, in which peptide peaks for further MS/MS identification are labeled out with mass value. D: The amino acid sequences of CLU, in which MS/MS matched peptide sequences are underlined.

To smooth intrinsic individual differences and enhance common characteristic traits only related to disease status, plasma samples from individuals in the same group were pooled together for the analysis. The same group of sample was labeled with either CyDye3 or CyDye5 and ran in two different gels (Additional file 1, Table S1). Dye swap images of each sample were acquired and analyzed to confirm the reproducible spot pattern with both dyes (Additional file 1, Figure S1). The 2-DE DIGE images of the samples of different groups labeled with different cyanine dyes were obtained by fluorescence scanning (Figure 1). The 2-D DIGE images were analyzed by DeCyder 5.0 to objectively estimate the abundance of proteins in each sample and to generate quantitative data. In total, 812 protein spots were auto-detected. Based on the threshold of |ratio| ≥ 1.5 and *P *≤ 0.05 (one-way ANOVA), the software detected 30 protein spots that showed a significant change among various groups (Figure 1 and Table 3). Figure 2 and 3 showed the identification of Prx II and CLU, respectively. Identification of Prx II by MALDI-TOF MS/MS is shown in Additional file 2, Figure S2. The raw MS data may be downloaded from the ProteomeCommons.org Tranche network using the following hash: TIa/TaABaUPU2yCPt/TxF8XDiKybQOSEqzWw76bpSSYwhmeMym9wDrfh+D4HYSudr1P3l9LQoH3RtcORcGfoaDvEh5w AAAAAAABCjQ==. The URL of this dataset is https://proteomecommons.org/dataset.jsp?i=TIa%2FTaABaUPU2yCPt%2FTxF8XDiKybQOSEqzWw76bpSSYwhmeMym9wDrfh%2BD4HYSudr1P3l9LQoH3RtcORcGfoaDvEh5wAAAAAAABCjQ%3D%3D, and of data download is https://proteomecommons.org/tranche/data-downloader.jsp?h=TIa%2FTaABaUPU2yCPt%2FTxF8XDiKybQOSEqzWw76bpSSYwhmeMym9wDrfh%2BD4HYSudr1P3l9LQoH3RtcORcGfoaDvEh5wAAAAAAABCjQ%3D%3D.

**Table 3:** List of identified proteins ^a^

**Spot No**.^**b)**^	**Protein ID**^**c)**^	**Procession No**.^**d)**^	**Protein Score**^**e)**^	**C.I.%**^**f)**^	**Ion Score**^**e)**^	**C.I.%**^**f)**^	Peptide	**MW(kDa)**^**g)**^ theoretical/observed	**pI**^**g)**^ theoretical/observed	**Appearance**^**h)**^	***P *value**^**I)**^
1	α-2 macroglobulin	P01023	341	100	185	100	34	163/90	6/5.6	12(18)	0.03
2	α-2 macroglobulin	P01023	341	100	185	100	34	163/90	6/5.7	12(18)	0.03
3	α-2 macroglobulin	P01023	341	100	185	100	34	163/90	6/5.8	12(18)	0.03
4	α-2 macroglobulin	P01023	341	100	185	100	34	163/90	6/5.9	12(18)	0.03
5	Serotransferrin	P02787	297	100	119	100	33	77/80	6.81/6.5	15(18)	0.012
6	Serotransferrin	P02787	78	99.992	37	99.6	13	77/80	6.81/6.5	12(18)	0.015
7	Hemopexin	P02790	120	100	44	99.996	19	52/60	6.55/4.5	15(18)	0.021
8	Hemopexin	P02790	178	100	80	100	19	52/60	6.55/4.5	15(18)	0.025
9	LRG	P02750	108	100	55	100	15	34/34	5.66/5.4	18(18)	0.029
10	LRG	P02750	199	100	134	100	18	34/34	5.66/5.4	18(18)	0.041
11	Fibrogen γ chain	P02679	146	100	40	99.987	18	50/50	5.61/5.9	15(18)	0.03
12	α-1 antitrypsin	P01009	76	99.986	33	99.942	11	47/56	5.37/5.2	12(18)	0.011
13	APO-AIV	P06727	143	100	41	99.991	15	45/45	5.28/5.2	18(18)	0.021
14	APO-AIV	P06727	143	100	41	99.991	15	45/45	5.28/5.2	18(18)	0.021
15	Fibrogen β chain	P02675	341	100	150	100	36	56/46	8.54/5.5	15(18)	0.021
16	Clusterin	P10909	144	100	86	100	17	52/40	5.89/5.0	18(18)	0.013
17	Clusterin	P10909	130	100	71	100	17	52/39	5.89/5.2	18(18)	0.013
18	Clusterin	P10909	74	99.98	59	100	12	52/35	5.89/5.3	18(18)	0.013
19	Clusterin	P10909	127	100	74	100	17	52/34	5.89/5.4	18(18)	0.016
20	APO-A1	P02647	255	100	87	100	28	31/28	5.56/5.2	18(18)	0.013
21	APO-A1	P02647	364	100	107	100	33	31/28	5.56/5.3	18(18)	0.013
22	APO-A1	P02647	214	100	21	99.305	25	31/28	5.56/5.3	18(18)	0.021
23	APO-A1	P02647	404	100	113	100	39	31/28	5.56/5.4	18(18)	0.021
24	Thioredoxin peroxidase 1	P32119	263	100	143	100	16	22/21	5.66/5.4	15(18)	0.021
25	HP-2	P00738	71	99.961	19	99.13	10	45/20	6.13/5.4	18(18)	0.0031
26	HP-2	P00737	91	100	58	100	12	38/20	6.13/5.6	18(18)	0.0051
27	HP-2	P00738	88	100	58	100	11	45/20	6.13/6.3	18(18)	0.0051
28	Prealbulmn	P02766	53	97.616	25	99.732	7	16/16	5.52/5.2	18(18)	0.0067
29	Prealbulmn	P02766	56	98.66	54	99.944	7	16/16	5.52/5.3	15(18)	0.0067
30	Prealbulmn	P02766	127	100	64	100	10	16/16	5.52/5.6	12(18)	0.013

a) Spots for which the volume |ratio| ≥ 1.5 (t test) and *P *≤ 0.05 (one-way ANOVA) based on DeCyder software analysis were identified by MALDI-TOF/TOF MS.

b) Spots referring to Figure 2.

c) Spots in the same line were identified as same protein.

d) Protein ID accessed from Swiss-Prot database by data searching.

e) Total protein score analyzed by MS and total ion score of the peptide analyzed by MS/MS.

f) Confidence of protein score and ion score.

g) Theoretical MW and pI accessed from UniProt database.

h) Number of the gels containing the protein spot.

i) *P *value calculated by one-way ANOVA analysis.

### Western blot analysis for Prx II and CLU in plasma

**Figure 4:**
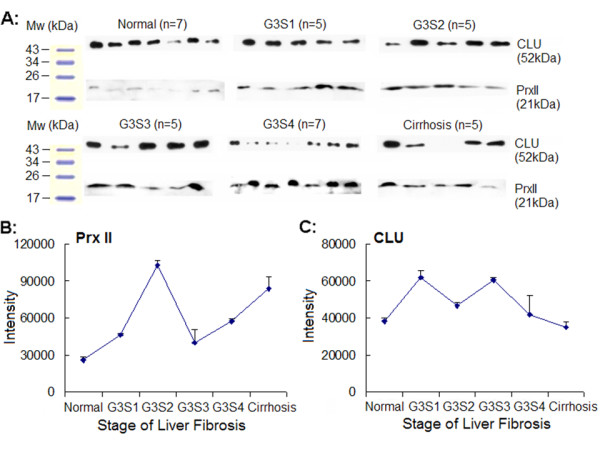
**Protein expressions of Prx II and CLU in plasma by Western blot analysis**. A: Single samples of normal and liver fibrosis. Immunoblotting with Prx II or CLU polyclonal antibody following SDS-PAGE was performed as described in Section 2.7. The films were scanned and the OD of each band in the film was evaluated by QuantitiOne software. B: The change of Prx II expression is similar to DIGE result (Figure 2.B). C: The change of CLU expression is similar to DIGE result (Figure 3.B).

To confirm the differential expression of Prx II, western blotting analysis was also performed using polyclonal antibodies against Prx II (Figure 4A). Because of the huge diversity of protein concentration in plasma among individuals, we did not normalize the result of western blot with a house-keeping protein as usual. Instead, we analyzed the expression of CLU from the same lane of each sample to avoid errors in sample loading and membrane transferring. As expected, the changes of both proteins were similar to that of DIGE result (Figure 4B,C), Compared to normal plasma, Prx II showed to be highly present in all stage of fibrosis plasma, although the up-regulation of Prx II was withdrawn at S3 stage. The presence of CLU showed to decrease continuously with the progress of fibrosis. Thus, the up-expression of Prx II was reliable.

### Measurement of Plasma Levels of Prx II and HA

**Figure 5:**
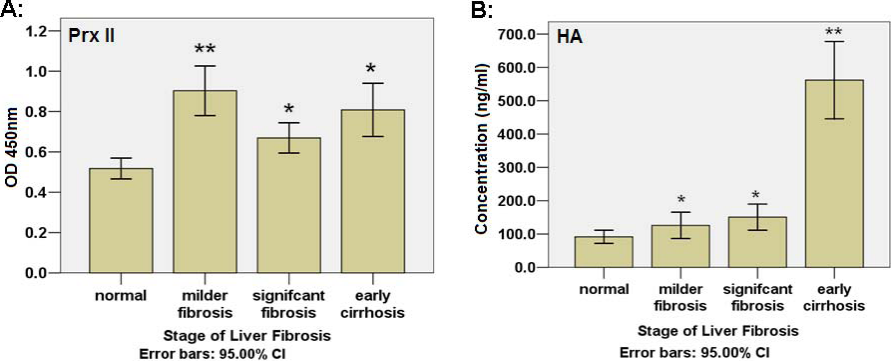
**Human plasma levels of Prx II and HA**. A: the plasma levels of Prx II were significantly elevated in patients with milder grade fibrosis compared to those in normal controls. The plasma level of Prx II in the late stage fibrosis and early cirrhosis were reduced compared to that in the early stage, but still higher than the normal controls. B: a significantly elevated HA plasma level among the early cirrhosis patients compared to normal controls and fibrosis patients. Compared to normal:* *P *< 0.05, ** *P *< 0.01

We observed a significantly increased level of plasma Prx II among the milder fibrosis patients (0.9033 ± 0.2925, n = 24) compared with that among the normal controls (0.5176 ± 0.1672, n = 42, *P *< 0.01). The plasma level of Prx II in the significant fibrosis (0.6681 ± 0.2090, n = 32) and early cirrhosis (0.8083 ± 0.2081, n = 12) were reduced compared to that in the early stage, but still higher than the normal controls (Figure 5A). We observed a significantly elevated level of plasma HA among the early cirrhosis patients (median = 561.9317 ± 183.0116, n = 12) compared to normal controls (median = 91.7035 ± 60.3199, n = 38, *P *< 0.01), milder fibrosis patients (125.8983 ± 93.3860, n = 24, *P *< 0.01) and significant fibrosis patients (150.6675 ± 108.6073, n = 32, *P *< 0.01) (Figure 5B).

### Clinical diagnosis of 25 serological analysis markers

Twenty-five serological analysis markers, including 4 differential proteins found by 2D-DIGE, 4 clinical plasma fibrosis markers and 17 serological biochemical markers were screened for their diagnosis values to various stages of fibrosis. Table 4 shows the areas under curves (AUCs) of discriminatory values of receiver operating characteristic (ROC) analysis of 25 serological markers to normal, milder fibrosis, significant fibrosis and early cirrhosis. For the discrimination of milder fibrosis, the area under curve (AUC) of Prx II was the largest (0.872 ± 0.090, Mean ± SD), higher than other 24 markers (0.148 ± 0.076~0.733 ± 0.098, Mean ± SD). For significant fibrosis, the AUC of CIV was the largest (0.799 ± 0.013), higher than other 24 markers (0.237 ± 0.076~0.750 ± 0.098, Mean ± SD). For early cirrhosis, the AUC of HA was the largest (1.000 ± 0.013), higher than other 24 markers (0.100 ± 0.076~0.907 ± 0.098, Mean ± SD). An AUC over 0.5 means the marker can be used for clinical diagnosis. The higher the AUC is, the more useful the marker may be. Unlike HA and CIV, which were useful for significant fibrosis and early stage cirrhosis diagnostician, Prx II was more efficient to milder fibrosis diagnostician.

**Table 4:** Areas under curve of ROC analysis of 25 serological markers

	**State Variable**^**b)**^			
**Test** **Variable**^**a)**^	**Normal**	**Milder fibrosis**	**Significant fibrosis**	**Early cirrhosis**

A	0.852	0.433	0.237	0.263
A/G	**0.898**	0.398	0.244	0.171
AFP	0.356	0.338	0.666	0.857
AKP	0.325	0.552	0.522	0.876
Apo AI	0.508	0.423	0.565	0.468
AST/ALT	0.818	0.148	0.247	0.692
CB	0.282	0.546	0.569	0.896
CHE	0.695	0.441	0.468	0.100
C-IV	0.136	0.470	**0.799**	0.894
CLU	0.290	0.527	0.738	0.453
G	0.119	0.610	0.713	0.878
HA	0.314	0.478	0.549	**1.0000**
HB	0.613	0.452	0.404	0.511
HP	0.329	0.601	0.603	0.537
LN	0.419	0.534	0.589	0.438
Prx II	0.216	**0.872**	0.500	0.577
PIIIP	0.475	0.512	0.498	0.558
PLT	0.512	0.426	0.372	0.226
Pre	0.543	0.506	0.568	0.263
PT	0.098	0.242	0.528	0.855
rGT	0.266	0.733	0.640	0.872
TB	0.3750	0.587	0.551	0.907
TC	0.688	0.424	0.294	0.637
TG	0.541	0.454	0.502	0.466
WBC	0.265	0.549	0.750	0.488

a) Abbreviations used for test variables

A: albumin; A/G: albumin/IgG; AFP: alpha fetal protein; AKP: alkaline phosphotase; AST: Aspartate aminotransferase; ALT: Alanine Aminotransferase; CB: Combined bilirubin; CHE: cholinesterase; C-IV: collagen type IV; CLU: clusterin; G: IgG; HA: Sodium Hyaluronate; HB: hemoglobin; HP: Haptoglobin; LN: laminin; Prx II: thioredoxin peroxidase 2; PIIIP: pre-collagen peptide type III; PLT: platelets; Pre: prealbumin; PT: prothrombin time; rGT: gama glutamyl transpeptidase; TB: total bilirubin; TC: total cholesterol; WBC: white blood cells

b) The maximal AUCs for each state are shown in bold.

**Figure 6:**
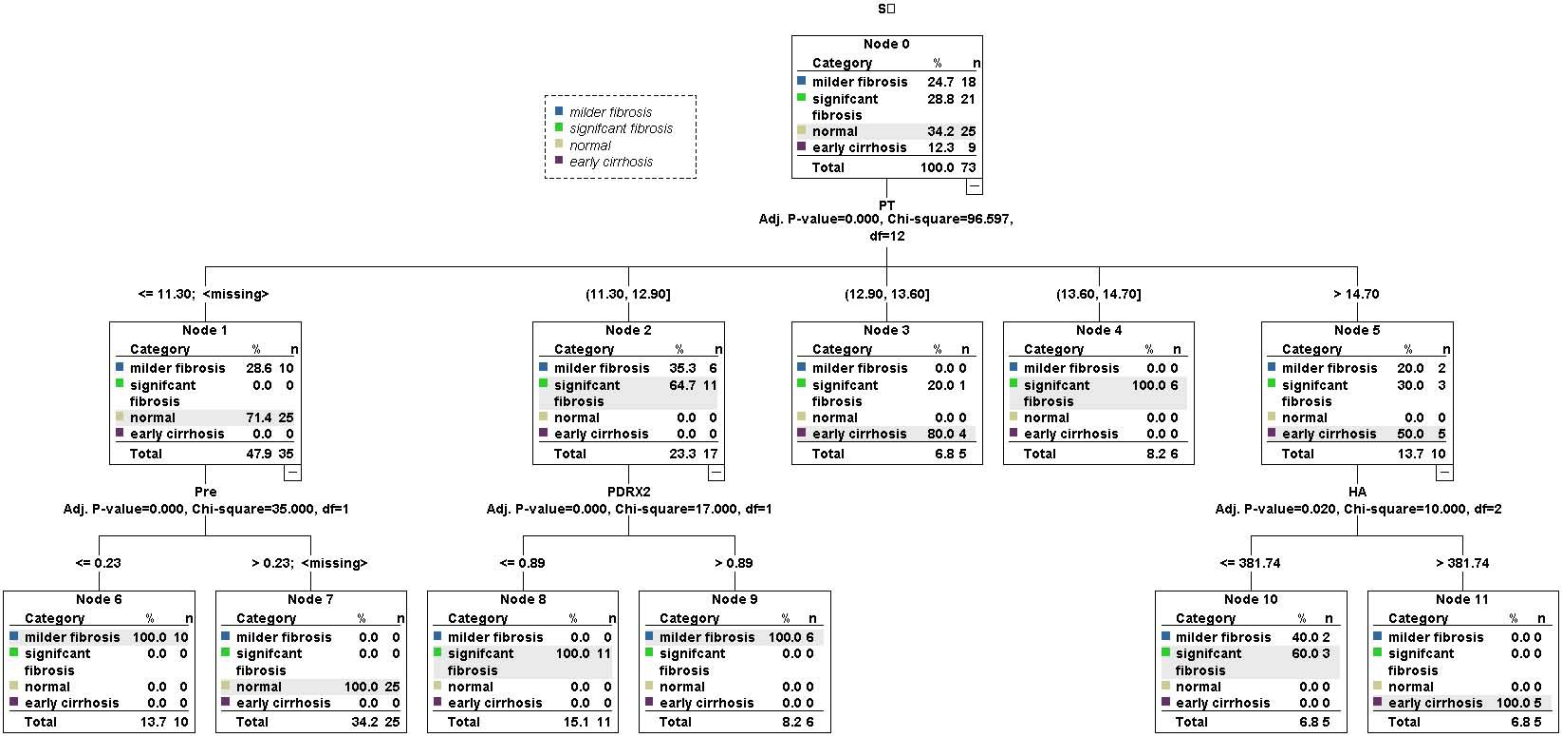
**Decision tree for the differentiation of various stages fibrosis using four serological markers**. Briefly, in a training group of 73 samples, samples were first divided into three groups (normal & milder fibrosis, milder & significant fibrosis, significant fibrosis & early cirrhosis) by marker "PT" (cutoff value = 12.00, 13.60 and 14.70, respectively); The normal & milder fibrosis group was then correctly classified by marker "Pre" (cutoff value = 0.23); the milder & significant fibrosis group was then correctly classified by marker "Prx II" (cutoff value = 0.80); the significant fibrosis & early cirrhosis group was then classified by marker "HA" (cutoff value = 381.74) and all the early cirrhosis samples (9/9) were correctly classified. The correct prediction percentage of the algorithm for normal control, milder fibrosis, significant fibrosis and early cirrhosis was 100%, 88.9%, 95.2% and 100%, respectively.

For differentiation between various grades fibrosis, four variables (PT, Pre, HA and Prx II) were selected by SPSS software from the 25 variables to construct a decision tree (Figure 6). In a training group, 73 samples were first divided into three groups by marker "PT" (cutoff value = 12.00, 13.60 and 14.70, respectively): normal (25/25) & milder fibrosis (10/18), milder (6/18) & significant fibrosis (11/21), significant fibrosis (10/21) & early cirrhosis (9/9). The normal & milder fibrosis group was then correctly classified by marker "Pre" (cutoff value = 0.23); the milder & significant fibrosis group was then correctly classified by marker "Prx II" (cutoff value = 0.80); the significant fibrosis & early cirrhosis group was then classified by marker "HA" (cutoff value = 381.74) and all the early cirrhosis samples (9/9) were correctly classified. The correct prediction percentage of the algorithm for normal control, milder fibrosis, significant fibrosis and early cirrhosis was 100%, 88.9%, 95.2% and 100%, respectively. The algorithm was further validated by a test group of 37 samples, and the correct prediction percentage for normal control, milder fibrosis, significant fibrosis and early cirrhosis was 100%, 100.0%, 88.9% and 100%, respectively (Table 5).

**Table 5:** Correct percentage of prediction for various grades fibrosis

		Predicted				
		
Sample Observed		normal	Milder fibrosis	Significant	Early cirrhosis	Percent Correct (%)
Training	normal	25	0	0	0	100
	milder fibrosis	0	16	2	0	88.9
	significant fibrosis	0	0	20	1	95.2
	early cirrhosis	0	0	0	9	100
	Overall Percentage	34.2	21.9	30.1	13.7	95.9
Test	normal	17	0	0	0	100
	milder fibrosis	0	6	0	0	100
	significant fibrosis	0	2	8	1	72.7
	early cirrhosis	0	0	0	3	100
	Overall Percentage	45.9	21.6	21.6	10.8	91.9

## Discussion

Plasma proteins are quite reflective of the overall profile in humans. It is estimated that as many as 10,000 proteins are present within human plasma, many of which are secreted or shed by cells during different physiology or pathology processes [24]. Moreover, instead of tissue, the utility of plasma to classify the disease state would have great advantages in that the plasma is easy to collect, the procedure is minimally invasive, and samples can be collected repeatedly. Therefore, considerable efforts have been made to discover plasma biomarkers for clinical purposes [25,26].

Recently, proteomics, a powerful strategy which can provide the global information of new biomarkers, disease associated targets and the process of pathogenesis by comprehensively examining different protein expression profiles between normal and pathological or drug treated samples, has been extensively employed to investigate cancers and other diseases. However, plasma proteome analysis is still a daunting task largely due to abundant proteins such as albumin and IgG that constitute approximately 60-97% of the total plasma proteins [27]. Efficient depletion of abundant proteins from human plasma enables the detection of more proteins with greater protein coverage [28]. On the other hand, the depletion of highly abundant proteins may result in the loss of potentially important proteins bound to them at the same time. The more kinds of abundant proteins are depleted, the more unspecific bounded proteins may loose [29]. Therefore, we chose to deplete albumin and IgG to minimize the unspecific protein depletion (Additional file 3, Figure S3). Depleting these two abundant proteins prior to the DIGE technology increased the loading volume from 5 μL to 35 μL (estimated raw plasma volumes with same protein amount), and significantly improved the detection of low abundant proteins. Proteins, such as clusterin, hemopexin and thioredoxin peroxidase, could be detected in the μg/mL range, which cannot be detected by the traditional 2-DE technology.

Pooling samples is a common way to reduce the cost of experiments as well as to provide equivalent power of experiments [30-32]. Since the purpose of our study is to identify robust biomarkers related with the progress of liver fibrosis, the differences among various groups are more interesting than the differences between patients within each patient group. We pooled the samples to smooth intrinsic individual differences and enhance common characteristic traits only related to disease status. It is also true that pooling samples may eliminate the number of biological replicates. Therefore, we analyzed protein levels in a larger population by ELISA to make up the disadvantages of pooling samples.

In this study, the albumin and IgG depletion strategy prior to 2-D DIGE was applied to enrich the low-abundant proteins in human plasma. By the 2-D DIGE, several proteins with significant alterations related with fibrosis progress were found. The up-regulated proteins were identified as fibrogen, collagen, macroglobulin, hemopexin, antitrypsin, prealbumin and thioredoxin peroxidase. The down-regulated proteins were haptoglobin, serotransferrin, CD5 antigen like protein, clusterin, apolipoprotein and leucine-rich alpha-2-glycoprotein (LRG). The biological functions of these proteins can be summarized into four groups: (A) Generation and degradation of extra cellular matrix (ECM), such as fibrogen, collagen and macroglobulin [33]; (B) Acute phase reaction and immunity protection, such as antitrypsin, prealbumin, LRG [34] and CD5 antigen-like protein; (C) Oxygenation and cell apoptosis, such as clusterin [35] and thioredoxin peroxidase [36,37]; (D) Transport and metabolism, such as apolipoprotein, haptoglobin, hemopexin and serotransferrin. As expected, proteins related to the generation of extra cellular matrix had the same alteration pattern of increase, which is in accordance with the progress of fibrosis. Proteins with the function of transport and metabolism had the same alteration pattern of decrease, which implied the dysfunction of the liver with the development of fibrosis. Most of the proteins found to be up- or down regulated were described in prior papers, which imply the reliability of our study design and the DIGE technology.

However, some of the identified proteins had different observed MW or pI, compared to theoretical ones. This is because that the proteins detected in plasma are secreted proteins, which are usually smaller than the whole protein. And the post translation modification of proteins, either carbamidomethyl, oxidation or phosphorylation, will change the pI of proteins greatly.

Of the 13 identified proteins, thioredoxin peroxidase appears to be a novel candidate as useful HBV-related milder grade liver fibrosis marker. Large evidence shows that in humans and animals, oxidative stress is implicated in the resistance to HBV infection and serves as a link between hepatic injury and fibrosis [38,39]. Thioredoxin peroxidases, also called peroxiredoxins, are members of a newly discovered family of peroxidases, and they efficiently reduced the intracellular level of H_2_O_2 _produced in those cells stimulated by various cell surface ligands. The peroxiredoxin family was reported to be closely related to various causes of liver fibrosis. They were found to be up-regulated in liver fibrosis caused by alcohol exposition [40], schistosomiasis [41], drug and chemical induction [41,42]. The oxidation kinetics of all peroxiredoxins was extremely rapid and sensitive, occurring at H_2_O_2 _doses unable to affect common markers of cellular oxidative stress [43]. In our research, Prx II has shown a significant up-regulation at the milder stage fibrosis, which indicated that it is an early protein target of HBV induced oxidative injury. On the contrary, current available fibrosis biomarkers, such as HA, CIV, PIIIP and LN, rely on the measurement of substances that participate in the generation of the liver extra cellular matrix and thus have limited clinical application value in milder fibrosis prediction.

As the complexity of liver fibrosis disallows any single biomarker to guide the diagnosis, prognosis, and treatment of the disease, we tried to use the tree classification system to predict the various fibrosis stages. Among the 25 serological analysis markers screened, PT, Pre, Prx II and HA were selected to construct a decision tree. The correct prediction percentages in both the training group and the test group were high. In the algorithm, Pre-albumin was used to correctly classify between normal and milder fibrosis, indicating an acute phase reaction at the beginning of fibrosis. Prx II was used to correctly classify between milder and significant fibrosis, indicating an anti-oxidative stress reaction during the progress of fibrosis. HA was used to correctly classify between significant fibrosis and early cirrhosis, indicating an assembling of ECM at the late stage of fibrosis.

## Conclusion

In this study, we have shown the quantitative plasma protein profiles in various stages liver fibrosis patients, and have found several proteins that changed significantly during disease progression. The differential expressed proteins have four groups of biological functions, which is helpful for revealing the underlying mechanisms of liver fibrosis. The significant up-regulation of Prx II implied that it held comparable sensitivity and specificity in the prediction of milder fibrosis, which may be useful for early fibrosis diagnosis if validated in other cohorts.

## Abbreviations

HBV: hepatitis B virus; HCC: hepatocellular carcinoma; 2-DE: two-dimensional electrophoresis; 2-D DIGE: two-dimensional differential in-gel electrophoresis; ELISA: enzyme linked immunosorbent assay; ANOVA: analysis of variance; ROC: receiver operating characteristic; AUC: area under curve; Prx II: thioredoxin peroxidase; CLU: clusterin; LRG: leucine-rich alpha-2-glycoprotein; HA: hyaluronic acid; CIV: type IV collagen; PIIIP: N-terminal propeptide of type III procollagen; HP: haptoglobin; LN: laminin; Apo AI: apolipoprotein AI; ECM: extra cellular matrix; TNF-a: tumour necrosis factor alpha; PDGF: platelet-derived growth factor

## Competing interests

The authors declare that they have no competing interests.

## Authors' contributions

YL carried out the 2D-DIGE experiments, protein identification, participated in the data analysis and drafted the manuscript. JL carried out the serological analysis and participated in the statistical analysis. CL carried out the western blot analysis and participated in the data analysis. HW and YJ participated in the design of the study and performed the statistical analysis. JW, PY and FH conceived of the study, participated in its design and coordination and helped to draft the manuscript. All authors read and approved the final manuscript.

## Pre-publication history

The pre-publication history for this paper can be accessed here:

http://www.biomedcentral.com/1471-230X/10/115/prepub

## Supplementary Material

Additional file 1 **Experiment design of DIGE**. Additional file 1 contains Table S1, in which the experiment design of DIGE is explained, and Figure S1, in which DIGE image of sample G3S1 labeled with Cy3 and Cy5 is shown. It can be seen that the spot pattern is reproducible with both dyes. Therefore, each sample was labeled with either Cy3 or Cy5 and ran on two individual gels to eliminate the possible effect of different Dyes on sample electrophoresis. Click here for file

Additional file 2 **Identification of Prx II by MALDI-TOF MS/MS**. Additional file 2 contains Figure S2, in which MALDI-TOF MS map of Prx II, tandem MS/MS spectra of peptide m/z 1211.71, 1735.02 and 1864.11, and the amino acid sequences of Prx II are shown. Click here for file

Additional file 3 **Effect of albumin and IgG depletion**. Additional file 3 contains Figure S3, in which 2-DE gels of raw plasma and depleted plasma are shown. It can be seen that after depletion, two most abundant plasma proteins, albumin and IgG, are successfully removed. Click here for file

## References

[B1] Oberti F Valsesia E Pilette C Rousselet MC Bedossa P Aube C Gallois Y Rifflet H Maiga MY Penneau-Fontbonne D Cales P Noninvasive diagnosis of hepatic fibrosis or cirrhosis Gastroenterology 1997 113 1609 1616 10.1053/gast.1997.v113.pm9352863 9352863

[B2] Wai CT Greenson JK Fontana RJ Kalbfleisch JD Marrero JA Conjeevaram HS Lok AS-F A simple noninvasive index can predict both significant fibrosis and cirrhosis in patients with chronic hepatitis C Hepatology 2003 38 518 526 10.1053/jhep.2003.50346 12883497

[B3] Guéchot J Poupon RE Giral P Balkau B Giboudeau J Poupon R Relationship between procollagen III amino terminal propeptide and hyaluronan serum levels and histological fibrosis in primary biliary cirrhosis and chronic viral hepatitis C J Hepatol 1994 20 388 393 10.1016/S0168-8278(94)80013-8 8014451

[B4] dos Santos VN Leite-Mór MMB Kondo M Martins JR Nader H Lanzoni VP Parise ER Serum laminin, type IV collagen and hyaluronan as fibrosis markers in non-alcoholic fatty liver disease Braz J Med Biol Res 2005 38 747 753 15917956 10.1590/s0100-879x2005000500012

[B5] Guechot J Laudat A Loria A Serfaty L Poupon R Giboudeau J Diagnostic accuracy of hyaluronan and type III procollagen amino- terminal peptide serum assays as markers of liver fibrosis in chronic viral hepatitis C evaluated by ROC curve analysis Clin Chem 1996 42 558 563 8605673

[B6] El-Gindy I El Rahman AT El-Alim MA Zaki SS Diagnostic potential of serum matrix metalloproteinase-2 and tissue inhibitor of metalloproteinase-1 as non-invasive markers of hepatic fibrosis in patients with HCV related chronic liver disease Egypt J Immunol 2003 10 27 35 15719620

[B7] Wang H Mengsteab S Tag CG Gao CF Hellerbrand C Lammert F Gressner AM Weiskirchen R Transforming growth factor-β1 gene polymorphisms are associated with progression of liver fibrosis in Caucasians with chronic hepatitis C infection World J Gastroenterol 2005 11 1929 1936 15800982 10.3748/wjg.v11.i13.1929 PMC4305713

[B8] Kim J Kim SH Lee SU Ha GH Kang DG Ha NY Ahn JS Cho HY Kang SJ Lee YJ Hong SC Ha WS Bae JM Lee CW Kim JW Proteome analysis of human liver tumor tissue by two-dimensional gel electrophoresis and matrixassisted laser desorption/ionization-mass spectrometry for identification of disease-related proteins Electrophoresis 2002 23 4142 4156 10.1002/elps.200290032 12481271

[B9] Chen G Gharib TG Huang CC Thomas DG Shedden KA Taylor JM Kardia SL Misek DE Giordano TJ Iannettoni MD Orringer MB Hanash SM Beer DG Proteomic Analysis of Lung Adenocarcinoma: Identification of a Highly Expressed Set of Proteins in Tumors Clin Cancer Res 2002 8 2298 2305 12114434

[B10] Meehan KL Holland JW Dawkins HJ Proteomic analysis of normal and malignant prostate tissue to identify novel proteins lost in cancer Prostate 2002 50 54 63 10.1002/pros.10032 11757036

[B11] Wulfkuhle JD Sgroi DC Krutzsch H McLean K McGarvey K Knowlton M Chen S Shu H Sahin A Kurek R Wallwiener D Merino MJ Petricoin EF III Zhao YM Steeg PS Proteomics of Human Breast Ductal Carcinoma in *Situ* Cancer Res 2002 62 6740 6749 12438275

[B12] Klade CS Voss T Krystek E Ahorn H Zatloukal H Pummer K Adolf GR Identification of tumor antigens in renal cell carcinoma by serological proteome analysis Proteomics 2001 1 890 898 10.1002/1615-9861(200107)1:7<890::AID-PROT890>3.0.CO;2-Z 11503213

[B13] He QY Chen J Kung HF Yuen APW Chiu JF Identification of tumor-associated proteins in oral tongue squamous cell carcinoma by proteomics Proteomics 2004 4 271 278 10.1002/pmic.200300550 14730689

[B14] Chen J He QY Yuen AP Chiu JF Proteomics of buccal squamous cell carcinoma: The involvement of multiple pathways in tumorigenesis Proteomics 2004 4 2465 2475 10.1002/pmic.200300762 15274141

[B15] Zhang LY Ying WT Mao TS He HZ Liu Y Wang HX Liu F Wang K Zhang DC Wang Y Wu M Qian XH Zhao XH Loss of clusterin both in serum and tissue correlates with the tumorigenesis of esophageal squamous cell carcinoma via proteomics approaches World J Gastroenterol 2003 4 650 654 10.3748/wjg.v9.i4.650 PMC4611421 12679903

[B16] Moreira JM Gromov P Celis JE Expression of the tumor suppressor protein 14-3-3σ is down regulated in invasive transitional cell carcinoma of the urinary bladder undergoing epithelial mesenchymal transition Mol Cell Proteomics 2004 3 410 419 10.1074/mcp.M300134-MCP200 14736829

[B17] Srisomsap C Sawangareetrakul P Subhasitanont P Panichakul T Keeratichamroen S Lirdprapamongkol K Chokchaichamnankit D Sirisinha S Svasti J Proteomic analysis of cholangiocarcinoma cell line Proteomics 2004 4 1135 1144 10.1002/pmic.200300651 15048994

[B18] O'Farrell PH High resolution two-dimensional electrophoresis of proteins J Biol Chem 1975 250 4007 4021 236308 PMC2874754

[B19] Unlu M Morgan ME Minden JS Two-Dimensional Electrophoresis Difference gel electrophoresis. A single gel method for detecting changes in protein extracts Electrophoresis 1997 18 2071 2077 10.1002/elps.1150181133 9420172

[B20] Wayne FP Detection technologies in proteome analysis J of Chromatography B 2002 771 3 31 10.1016/S1570-0232(02)00043-0 12015990

[B21] Willard MF Scott EH Proteomics for protein expression profiling in neuroscience Neurochemical Research 2004 29 1065 1081 10.1023/B:NERE.0000023594.21352.17 15176464 PMC3843356

[B22] Desmet VJ Gerber M Hoofnagle JH Manns M Scheuer P Classification of Chronic Hepatitis: Diagnosis, Grading and Staging J Hepatology 1994 19 1513 1520 10.1002/hep.1840190629 8188183

[B23] Lv S Wei L Wang JH Wang JY Liu F Identification of Novel Molecular Candidates for Acute Liver Failure in Plasma of BALB/c Murine Model J Proteome Res 2007 6 7 2746 2752 10.1021/pr0701759 17569552

[B24] Anderson NL Anderson NG The Human Plasma Proteome: History, Character, and Diagnostic Prospects Mol Cell Proteomics 2002 1 845 867 10.1074/mcp.R200007-MCP200 12488461

[B25] Xu A Wang Y Xu JY Stejskal D Tam S Zhang J Wat NM Wong WK Lam KS Adipocyte Fatty Acid-Binding Protein Is a Plasma Biomarker Closely Associated with Obesity and Metabolic Syndrome Clin Chem 2006 52 3 405 413 10.1373/clinchem.2005.062463 16423904

[B26] Schorge JO Drake RD Lee H Skates SJ Rajanbabu R Miller DS Kim JH Cramer DW Berkowitz RS Mok SC Osteopontin as an Adjunct to CA125 in Detecting Recurrent Ovarian Cancer Clin Cancer Res 2004 10 10 3474 3478 10.1158/1078-0432.CCR-03-0365 15161704

[B27] Putnam RWthe Plasma Proteins 1975 Academic Press, New York 23 34

[B28] Tang HY Ali-Khan N Echan LA Levenkova N Rux JJ Speicher DW A novel four-dimensional strategy combining protein and peptide separation methods enables detection of low-abundance proteins in human plasma and serum proteomes Proteomics 2005 5 3329 3342 10.1002/pmic.200401275 16052622

[B29] Echan LA Tang HY Ali-Khan N Lee K Speicher DW Depletion of multiple high-abundance proteins improves protein profiling capacities of human serum and plasma Proteomics 2005 5 3292 3303 10.1002/pmic.200401228 16052620

[B30] Kendziorski C Irizarry RA Chen KS Haag JD Gould MN On the utility of pooling biological samples in microarray experiments Proc Natl Acad Sci USA 2005 102 4252 4257 10.1073/pnas.0500607102 15755808 PMC552978

[B31] Kendziorski CM Zhang Y Lan H Attie AD The efficiency of pooling mRNA in microarray experiments Biostatistics 2003 4 465 477 10.1093/biostatistics/4.3.465 12925512

[B32] Peng X Wood CL Blalock EM Chen KC Landfield PW Stromberg AJ Statistical implications of pooling NA samples for microarray experiments BMC Bioinformatics 2003 4 26 10.1186/1471-2105-4-26 12823867 PMC166151

[B33] Arthur MJ Degradation of matrix proteins in liver fibrosis Pathology research and practice 1994 190 825 833 10.1016/S0344-0338(11)80985-4 7899131

[B34] Li X Miyajima M Mineki R Taka H Murayama K Arai H Analysis of potential diagnostic biomarkers in cerebrospinal fluid of idiopathic normal pressure hydrocephalus by proteomics Acta Neurochir (Wien) 2006 148 859 864 10.1007/s00701-006-0787-4 16755327

[B35] Janig E Stumptner C Fuchsbichler A Denk H Zatloukal K Interaction of stress proteins with misfolded keratins Eur J Cell Biol 2005 84 329 39 10.1016/j.ejcb.2004.12.018 15819411

[B36] Chae HZ Robison K Poole LB Church G Storz G Rhee SG Cloning and sequencing of thiol-specific antioxidant from mammalian brain: alkyl hydroperoxide reductase and thiol-specific antioxidant define a large family of antioxidant enzymes Proc Natl Acad Sci USA 1994 91 7017 7021 10.1073/pnas.91.15.7017 8041738 PMC44329

[B37] Hofmann B Hecht HJ Flohe L Peroxideroxins Biol Chem 2002 383 347 364 10.1515/BC.2002.040 12033427

[B38] Shimizu I Recent Therapeutic Developments in Hepatic Fibrosis Current Drug Targets Infectious Disorders 2001 1 227 240 10.2174/1568005014606053 12455417

[B39] Shimizu I Impact of oestrogens on the progression of liver disease Liver International 2003 23 63 69 10.1034/j.1600-0676.2003.00811.x 12640729

[B40] Kim BJ Hood BL Aragon RA Hardwick JP Conrads TP Veenstra TD Song BJ Increased oxidation and degradation of cytosolic proteins in alcohol-exposed mouse liver and hepatoma cells Proteomics 2006 6 1250 1260 10.1002/pmic.200500447 16408314 PMC1368983

[B41] Meneses-Lorente G Guest PC Lawrence J Muniappa N Knowles MR Skynner HA Salim K Cristea I Mortishire-Smith R Gaskell SJ Watt A A Proteomic Investigation of Drug-Induced Steatosis in Rat Liver Chem Res Toxicol 2004 17 605 612 10.1021/tx034203n 15144217

[B42] Heijne WH Slitt AL van Bladeren PJ Groten JP Klaassen CD Stierum RH van Ommen B Bromobenzene-Induced Hepatotoxicity at the Transcriptome Level Toxicol Sci 2004 79 411 422 10.1093/toxsci/kfh128 15056800

[B43] Zhang W Wang M Xie HY Zhou L Meng XQ Shi J Zheng S Role of Reactive Oxygen Species in Mediating Hepatic Ischemia-Reperfusion Injury and Its Therapeutic Applications in Liver Transplantation Transplant Proc 2007 39 1332 1337 10.1016/j.transproceed.2006.11.021 17580134

