# Supplementary material for: Peroxiredoxin 2: a potential biomarker for early diagnosis of Hepatitis B Virus related liver fibrosis identified by proteomic analysis of the plasma
Source: BMC Gastroenterol. 2010 Oct 13;10:115. doi: 10.1186/1471-230X-10-115 (PMC2959091; doi:10.1186/1471-230X-10-115)

**Table S1** Experiment design of DIGE. Each sample was labeled with either Cy3 or Cy5 and ran on two individual gels to eliminate the possible effect of different Dyes on sample electrophoresis.

|  | Cy3 | Cy5 | Cy2 |
| --- | --- | --- | --- |
| Gel 1 | Normal | Cirrhosis | Internal Standard |
| Gel 2 | G3S1 | G3S4 | Internal Standard |
| Gel 3 | G3S2 | G3S3 | Internal Standard |
| Gel 4 | G3S3 | G3S2 | Internal Standard |
| Gel 5 | G3S4 | G3S1 | Internal Standard |
| Gel 6 | Cirrhosis | Normal | Internal Standard |

**Figure S1** DIGE image of sample G3S1 labeled with Cy3 and Cy5. It can be seen that the spot pattern is reproducible with both dyes.


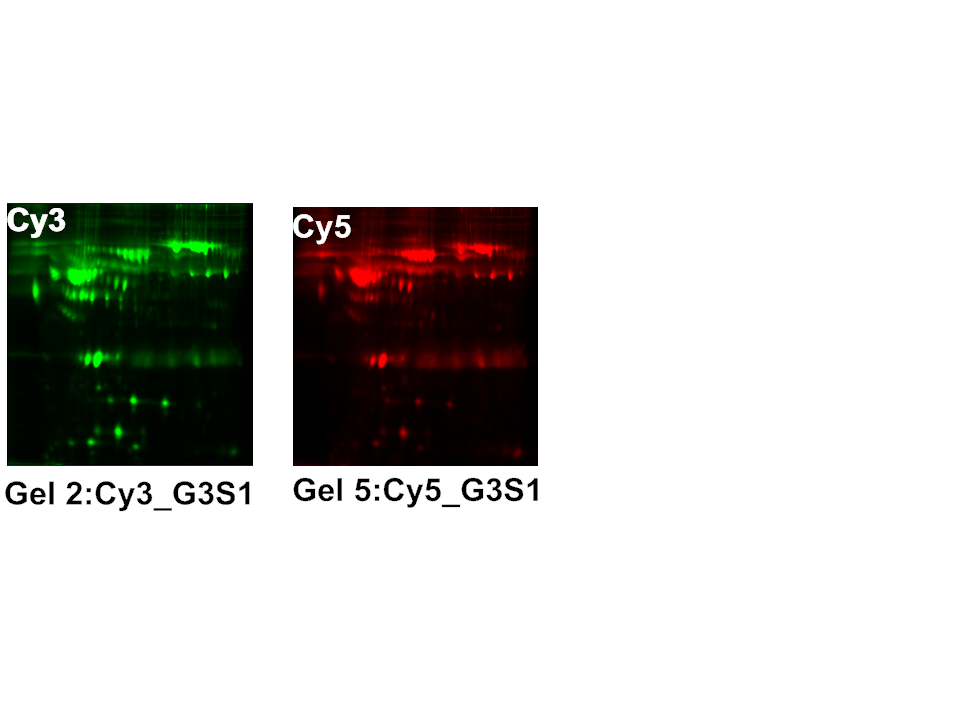

Supplement: Additional file 1 — Experiment design of DIGE. Additional file 1 contains Table S1, in which the experiment design of DIGE is explained, and Figure S1, in which DIGE image of sample G3S1 labeled with Cy3 and Cy5 is shown. It can be seen that the spot pattern is reproducible with both dyes. Therefore, each sample was labeled with either Cy3 or Cy5 and ran on two individual gels to eliminate the possible effect of different Dyes on sample electrophoresis. [file 1471-230X-10-115-S1.DOC]
