# Supplementary material for: Peroxiredoxin 2: a potential biomarker for early diagnosis of Hepatitis B Virus related liver fibrosis identified by proteomic analysis of the plasma
Source: BMC Gastroenterol. 2010 Oct 13;10:115. doi: 10.1186/1471-230X-10-115 (PMC2959091; doi:10.1186/1471-230X-10-115)

**Figure S2** Identification of Prx II by MALDI-TOF MS/MS. (A) shows the MALDI-TOF MS map of Prx II, in which peptide peaks for further MS/MS identification are labeled out with mass value. (B), (C) and (D) show the tandem MS/MS spectra of peptide m/z 1211.71, 1735.02 and 1864.11, respectively. (E) shows the amino acid sequences of Prx II, in which matched peptide sequences are underlined.


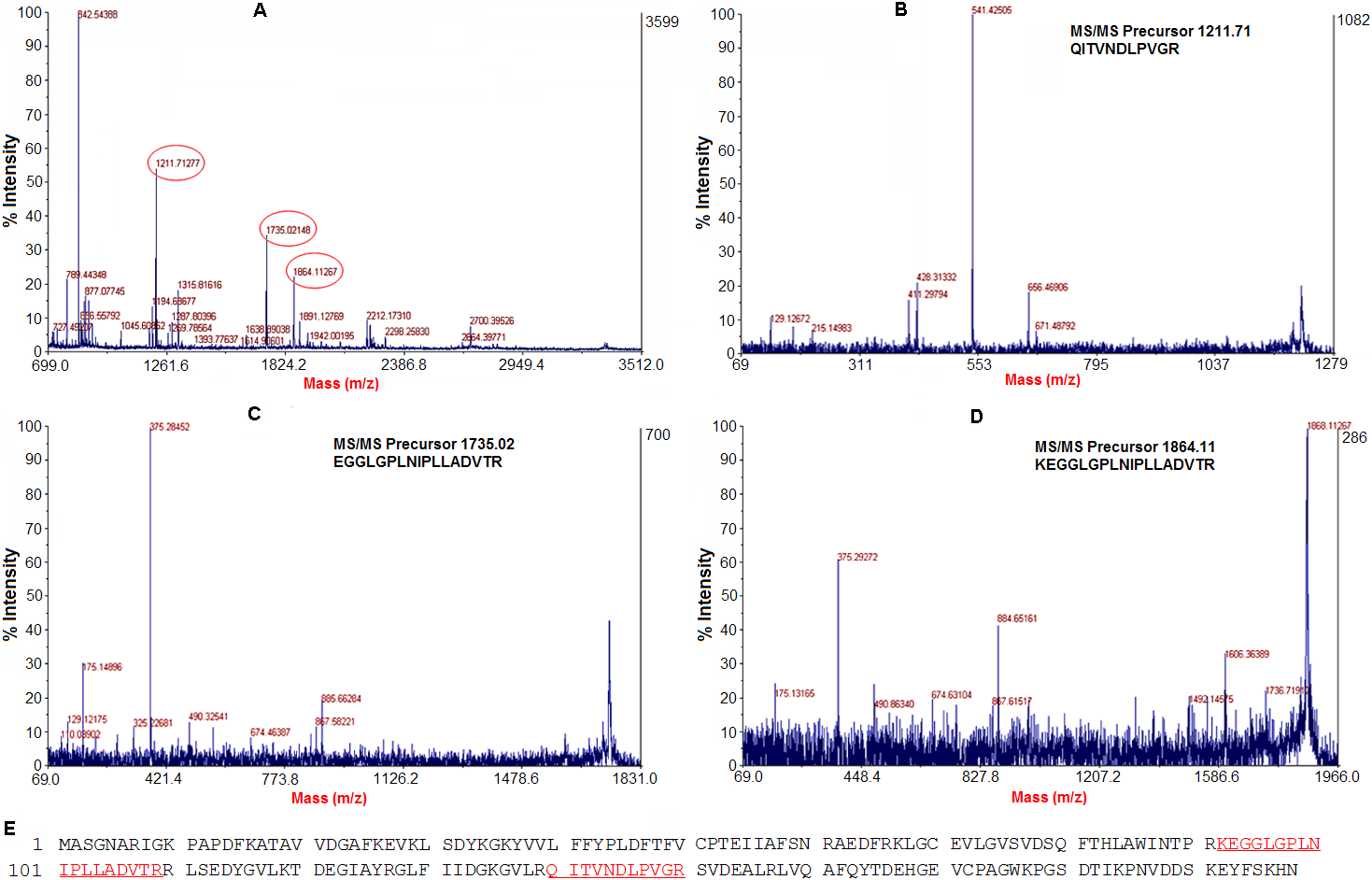

Supplement: Additional file 2 — Identification of Prx II by MALDI-TOF MS/MS. Additional file 2 contains Figure S2, in which MALDI-TOF MS map of Prx II, tandem MS/MS spectra of peptide m/z 1211.71, 1735.02 and 1864.11, and the amino acid sequences of Prx II are shown. [file 1471-230X-10-115-S2.DOC]
