# Supplementary material for: Peroxiredoxin 2: a potential biomarker for early diagnosis of Hepatitis B Virus related liver fibrosis identified by proteomic analysis of the plasma
Source: BMC Gastroenterol. 2010 Oct 13;10:115. doi: 10.1186/1471-230X-10-115 (PMC2959091; doi:10.1186/1471-230X-10-115)

**Figure S3** Effect of albumin and IgG depletion. (A) 2-DE gel of raw plasma. (B) 2-DE gel of depleted plasma. After depletion, two most abundant plasma proteins, albumin and IgG, are successfully removed.


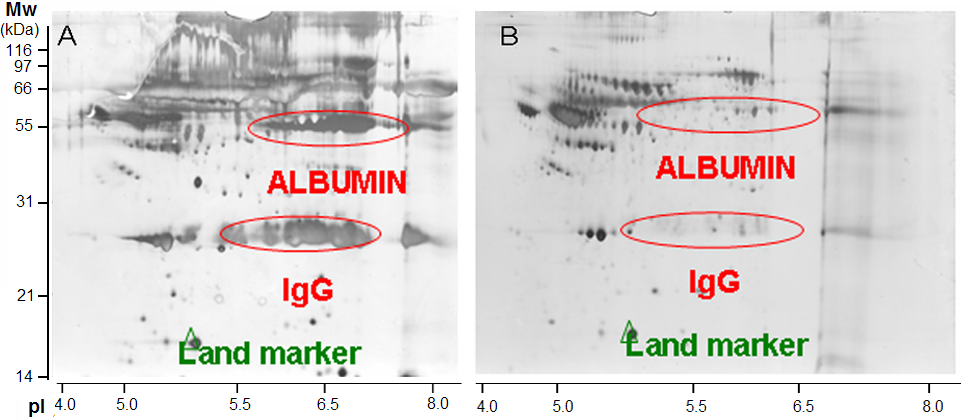

Supplement: Additional file 3 — Effect of albumin and IgG depletion. Additional file 3 contains Figure S3, in which 2-DE gels of raw plasma and depleted plasma are shown. It can be seen that after depletion, two most abundant plasma proteins, albumin and IgG, are successfully removed. [file 1471-230X-10-115-S3.DOC]
